# Supplementary material for: Are carbon emissions trading and green financial instruments synergistic? -Comprehensive quantitative research based on content analysis
Source: PLoS One. 2024 Mar 7;19(3):e0298601. doi: 10.1371/journal.pone.0298601 (PMC10919601; doi:10.1371/journal.pone.0298601)
Supplement: S1 Data — (ZIP) [file pone.0298601.s001.zip › Data/地级市Z_F工作报告.zip!/╕╩╦α/╝╬╙°╣╪╩╨/└┤╫╘╣1⁄2╓┌║┼í╛┬φ┐╦╩2╛▌═°í┐_2016.docx]

各位代表：

　　现在，我代表国务院，向大会报告政府工作，请予审议，并请全国政协各位委员提出意见。

　　一、2015年工作回顾

　　过去一年，我国发展面临多重困难和严峻挑战。在以习近平同志为总书记的党中央坚强领导下，全国各族人民以坚定的信心和非凡的勇气，攻坚克难，开拓进取，经济社会发展稳中有进、稳中有好，完成了全年主要目标任务，改革开放和社会主义现代化建设取得新的重大成就。

　　——经济运行保持在合理区间。国内生产总值达到67.7万亿元，增长6.9%，在世界主要经济体中位居前列。粮食产量实现"十二连增"，居民消费价格涨幅保持较低水平。特别是就业形势总体稳定，城镇新增就业1312万人，超过全年预期目标，成为经济运行的一大亮点。

　　——结构调整取得积极进展。服务业在国内生产总值中的比重上升到50.5%，首次占据"半壁江山"。消费对经济增长的贡献率达到66.4%。高技术产业和装备制造业增速快于一般工业。单位国内生产总值能耗下降5.6%。

　　——发展新动能加快成长。创新驱动发展战略持续推进，互联网与各行业加速融合，新兴产业快速增长。大众创业、万众创新蓬勃发展，全年新登记注册企业增长21.6%，平均每天新增1.2万户。新动能对稳就业、促升级发挥了突出作用，正在推动经济社会发生深刻变革。

　　——人民生活进一步改善。全国居民人均可支配收入实际增长7.4%，快于经济增速。去年末居民储蓄存款余额增长8.5%，新增4万多亿元。又解决6434万农村人口饮水安全问题。扶贫攻坚力度加大，农村贫困人口减少1442万人。

　　科技领域一批创新成果达到国际先进水平，第三代核电技术取得重大进展，国产C919大型客机总装下线，屠呦呦获得诺贝尔生理学或医学奖。对我国发展取得的成就，全国各族人民倍感振奋和自豪！

　　回顾过去一年，成绩来之不易。这些成绩，是在极为复杂严峻的国际环境中取得的。去年世界经济增速为6年来最低，国际贸易增速更低，大宗商品价格深度下跌，国际金融市场震荡加剧，对我国经济造成直接冲击和影响。这些成绩，是在国内深层次矛盾凸显、经济下行压力加大的情况下取得的。面对"三期叠加"的局面，经济工作遇到不少两难甚至多难问题，需要远近结合，趋利避害，有效应对。这些成绩，是在我国经济总量超过60万亿元的高基数上取得的。现在国内生产总值每增长1个百分点的增量，相当于5年前1.5个百分点、10年前2.5个百分点的增量。经济规模越大，增长难度随之增加。在困难和压力面前，全国各族人民付出了极大辛劳，一步一步走了过来。这再次表明，任何艰难险阻都挡不住中国发展前行的步伐！

　　一年来，我们主要做了以下工作：

　　一是着力稳增长调结构防风险，创新宏观调控方式。为应对持续加大的经济下行压力，我们在区间调控基础上，实施定向调控和相机调控。积极的财政政策注重加力增效，扩大结构性减税范围，实行普遍性降费，盘活财政存量资金。发行地方政府债券置换存量债务3.2万亿元，降低利息负担约2000亿元，减轻了地方政府偿债压力。稳健的货币政策注重松紧适度，多次降息降准，改革存贷比管理，创新货币政策工具，加大对实体经济支持力度。扩大有效投资，设立专项基金，加强水利、城镇棚户区和农村危房改造、中西部铁路和公路等薄弱环节建设。实施重点领域消费促进工程，城乡居民旅游、网购、信息消费等快速增长。去年还积极应对金融领域的多种风险挑战，守住了不发生系统性区域性风险的底线，维护了国家经济金融安全。

　　二是围绕激发市场活力，加大改革开放力度。我们不搞"大水漫灌"式的强刺激，而是持续推动结构性改革。深入推进简政放权、放管结合、优化服务改革。取消和下放311项行政审批事项，取消123项职业资格许可和认定事项，彻底终结了非行政许可审批。工商登记前置审批精简85%，全面实施三证合一、一照一码。加强事中事后监管，优化公共服务流程。群众和企业办事更加方便，全社会创业创新热情日益高涨。

　　财税金融等重点改革深入推进。中央对地方专项转移支付项目减少三分之一，一般性转移支付规模增加。营改增稳步实施，资源税从价计征范围扩大。取消存款利率浮动上限，推出存款保险制度，建立人民币跨境支付系统。价格改革力度加大，中央政府定价项目减少80%，地方政府定价项目减少一半以上。国有企业、农村、投融资、生态文明等领域改革有序推进，全面深化改革的成效正在显现。

　　坚持以开放促改革促发展。努力稳定对外贸易，调整出口退税负担机制，清理规范进出口环节收费，提高贸易便利化水平，出口结构发生积极变化。外商投资限制性条目减少一半，95%以上实行备案管理，实际使用外资1263亿美元，增长5.6%。非金融类对外直接投资1180亿美元，增长14.7%。推广上海自贸试验区经验，新设广东、天津、福建自贸试验区。人民币加入国际货币基金组织特别提款权货币篮子。亚洲基础设施投资银行正式成立，丝路基金投入运营。签署中韩、中澳自贸协定和中国-东盟自贸区升级议定书。"一带一路"建设成效显现，国际产能合作步伐加快，高铁、核电等中国装备走出去取得突破性进展。

　　三是聚焦提质增效，推动产业创新升级。制定实施创新驱动发展战略纲要和意见，出台推动大众创业、万众创新政策举措，落实"互联网+"行动计划，增强经济发展新动力。一大批创客走上创业创新之路。完善农业支持政策，促进农业发展方式加快转变。针对工业增速下降、企业效益下滑，我们一手抓新兴产业培育，一手抓传统产业改造提升。启动实施《中国制造2025》，设立国家新兴产业创业投资引导基金、中小企业发展基金，扩大国家自主创新示范区。积极化解过剩产能，推进企业兼并重组。近三年淘汰落后炼钢炼铁产能9000多万吨、水泥2.3亿吨、平板玻璃7600多万重量箱、电解铝100多万吨。促进生产性、生活性服务业加快发展。狠抓节能减排和环境保护，各项约束性指标超额完成。公布自主减排行动目标，推动国际气候变化谈判取得积极成果。

　　四是着眼开拓发展空间，促进区域协调发展和新型城镇化。继续推动东、中、西、东北地区"四大板块"协调发展，重点推进"一带一路"建设、京津冀协同发展、长江经济带发展"三大战略"，在基础设施、产业布局、生态环保等方面实施一批重大工程。制定实施促进西藏和四省藏区、新疆发展的政策措施。推进户籍制度改革，出台居住证制度，加强城镇基础设施建设，新型城镇化取得新成效。

　　五是紧扣增进民生福祉，推动社会事业改革发展。在财力紧张情况下，保障民生力度继续加大。推出新的政策，重点解决高校毕业生和就业困难群体的就业创业问题。城镇保障性安居工程住房基本建成772万套，棚户区住房改造开工601万套，农村危房改造432万户，一大批住房困难家庭圆了安居梦。加快改善贫困地区义务教育薄弱学校办学条件，深化中小学教师职称制度改革，重点高校招收贫困地区农村学生人数又增长10.5%。全面推开县级公立医院综合改革，拓展居民大病保险，建立重特大疾病医疗救助制度、困难残疾人生活补贴和重度残疾人护理补贴制度。提高低保、优抚、企业退休人员基本养老金等标准，推行机关事业单位养老保险制度改革并完善工资制度。加强基本公共文化服务建设。广大人民群众有了更多获得感。

　　六是促进社会和谐稳定，推动依法行政和治理方式创新。国务院提请全国人大常委会审议法律议案11件，制定修订行政法规8部。政务公开加快推进，推广电子政务和网上办事。建立重大政策落实督查问责机制，开展第三方评估。有效应对自然灾害和突发事件。加强安全生产监管，事故总量和重特大事故、重点行业事故数量继续下降。推进食品安全创建示范行动。强化社会治安综合治理，依法打击各类违法犯罪活动，有力维护了公共安全。

　　我们深入开展"三严三实"专题教育，锲而不舍落实党中央八项规定精神，坚决纠正"四风"，严格执行国务院"约法三章"。加强行政监察和审计监督。大力推进党风廉政建设和反腐败斗争，一批腐败分子受到惩处。

　　我们隆重纪念中国人民抗日战争暨世界反法西斯战争胜利70周年，集中宣示了我国作为世界反法西斯战争东方主战场的历史地位和重大贡献，彰显了中国人民同各国人民共护和平、共守正义的坚定信念！

　　一年来，全方位外交成果丰硕。习近平主席等国家领导人出访多国，出席联合国系列峰会、二十国集团领导人峰会、亚太经合组织领导人非正式会议、气候变化大会、东亚合作领导人系列会议、世界经济论坛等重大活动。成功举行中非合作论坛峰会、中欧领导人会晤，启动中拉论坛。同主要大国关系取得新进展，同周边国家务实合作深入推进，同发展中国家友好合作不断拓展，同联合国等国际组织和国际机制的关系全面加强，经济外交、人文交流卓有成效。中国作为负责任大国，在国际和地区事务中发挥了重要的建设性作用。

　　各位代表！

　　过去一年取得的成绩，是以习近平同志为总书记的党中央统揽全局、科学决策的结果，是全党全军全国各族人民齐心协力、顽强拼搏的结果。我代表国务院，向全国各族人民，向各民主党派、各人民团体和各界人士，表示诚挚感谢！向香港特别行政区同胞、澳门特别行政区同胞、台湾同胞和海外侨胞，表示诚挚感谢！向关心和支持中国现代化建设事业的各国政府、国际组织和各国朋友，表示诚挚感谢！

　　在充分肯定去年成绩的同时，我们也清醒看到，我国发展中还存在不少困难和问题。受全球贸易萎缩等因素影响，去年我国进出口总额出现下降，预期增长目标未能实现。投资增长乏力，一些行业产能过剩严重，部分企业生产经营困难，地区和行业走势分化，财政收支矛盾突出，金融等领域存在风险隐患。人民群众关心的医疗、教育、养老、食品药品安全、收入分配、城市管理等方面问题较多，严重雾霾天气在一些地区时有发生。特别令人痛心的是，去年发生了"东方之星"号客轮翻沉事件和天津港特别重大火灾爆炸等事故，人员伤亡和财产损失惨重，教训极其深刻，必须认真汲取。政府工作还存在不足，有些改革和政策措施落实不到位，少数干部不作为、不会为、乱作为，一些领域的不正之风和腐败问题不容忽视。我们要进一步增强忧患意识和担当意识，下更大力气解决这些问题，始终以民之所望为施政所向，尽心竭力做好政府工作，决不辜负人民重托。

　　各位代表！

　　2015年是"十二五"收官之年。过去五年，我国发展成就举世瞩目。党的十八大以来，在以习近平同志为总书记的党中央坚强领导下，面对错综复杂的国际环境和艰巨繁重的国内改革发展稳定任务，我们继续坚持稳中求进工作总基调，深化改革开放，实施一系列利当前、惠长远的重大举措，"十二五"规划确定的主要目标任务全面完成。一是经济持续较快发展。国内生产总值年均增长7.8%，经济总量稳居世界第二位，成为全球第一货物贸易大国和主要对外投资大国。二是结构调整取得标志性进展。服务业成为第一大产业，工业化与信息化融合加深，农业综合生产能力明显增强。消费成为支撑经济增长的主要力量。超过一半人口居住在城镇。单位国内生产总值能耗下降18.2%，主要污染物排放量减少12%以上。三是基础设施水平全面跃升。铁路营业里程达到12.1万公里，其中高速铁路超过1.9万公里，占世界60%以上。高速公路通车里程超过12万公里。南水北调东、中线工程通水。建成全球最大的第四代移动通信网络。四是科技创新实现重大突破。量子通信、中微子振荡、高温铁基超导等基础研究取得一批原创性成果，载人航天、探月工程、深海探测等项目达到世界先进水平。五是人民生活水平显著提高。居民收入增长快于经济增长，城乡收入差距持续缩小。城镇新增就业人数超过6400万人。城镇保障性安居工程住房建设4013万套，上亿群众喜迁新居。农村贫困人口减少1亿多，解决3亿多农村人口饮水安全问题。六是社会发展成就斐然。教育公平和质量明显提升。基本医疗保险实现全覆盖，基本养老保险参保率超过80%。文化软实力持续提升。依法治国全面推进。中国特色军事变革成就显著。经过五年努力，我国经济实力、科技实力、国防实力、国际影响力又上了一个大台阶。

　　"十二五"时期的辉煌成就，充分显示了中国特色社会主义的巨大优越性，集中展现了中国人民的无穷创造力，极大增强了中华民族的自信心和凝聚力，必将激励全国各族人民在实现"两个一百年"奋斗目标的新征程上奋力前行！

　　二、"十三五"时期主要目标任务和重大举措

　　根据《中共中央关于制定国民经济和社会发展第十三个五年规划的建议》，国务院编制了《国民经济和社会发展第十三个五年规划纲要(草案)》，提交大会审查。

　　《纲要草案》紧紧围绕全面建成小康社会奋斗目标，针对发展不平衡、不协调、不可持续等突出问题，强调要牢固树立和贯彻落实创新、协调、绿色、开放、共享的发展理念，明确了今后五年经济社会发展的主要目标任务，提出了一系列支撑发展的重大政策、重大工程和重大项目，突出了以下六个方面。

　　——保持经济中高速增长，推动产业迈向中高端水平。实现全面建成小康社会目标，到2020年国内生产总值和城乡居民人均收入比2010年翻一番，"十三五"时期经济年均增长保持在6.5%以上。加快推进产业结构优化升级，实施一批技术水平高、带动能力强的重大工程。到2020年，先进制造业、现代服务业、战略性新兴产业比重大幅提升，全员劳动生产率从人均8.7万元提高到12万元以上。届时，我国经济总量超过90万亿元，发展的质量和效益明显提高。在我们这样一个人口众多的发展中国家，这将是非常了不起的成就。

　　——强化创新引领作用，为发展注入强大动力。创新是引领发展的第一动力，必须摆在国家发展全局的核心位置，深入实施创新驱动发展战略。启动一批新的国家重大科技项目，建设一批高水平的国家科学中心和技术创新中心，培育壮大一批有国际竞争力的创新型领军企业。持续推动大众创业、万众创新。促进大数据、云计算、物联网广泛应用。加快建设质量强国、制造强国。到2020年，力争在基础研究、应用研究和战略前沿领域取得重大突破，全社会研发经费投入强度达到2.5%，科技进步对经济增长的贡献率达到60%，迈进创新型国家和人才强国行列。

　　——推进新型城镇化和农业现代化，促进城乡区域协调发展。缩小城乡区域差距，既是调整经济结构的重点，也是释放发展潜力的关键。要深入推进以人为核心的新型城镇化，实现1亿左右农业转移人口和其他常住人口在城镇落户，完成约1亿人居住的棚户区和城中村改造，引导约1亿人在中西部地区就近城镇化。到2020年，常住人口城镇化率达到60%、户籍人口城镇化率达到45%。实施一批水利、农机、现代种业等工程，推动农业适度规模经营和区域化布局、标准化生产、社会化服务。到2020年，粮食等主要农产品供给和质量安全得到更好保障，农业现代化水平明显提高。以区域发展总体战略为基础，以"三大战略"为引领，形成沿海沿江沿线经济带为主的纵向横向经济轴带，培育一批辐射带动力强的城市群和增长极。加强重大基础设施建设，高铁营业里程达到3万公里、覆盖80%以上的大城市，新建改建高速公路通车里程约3万公里，实现城乡宽带网络全覆盖。

　　——推动形成绿色生产生活方式，加快改善生态环境。坚持在发展中保护、在保护中发展，持续推进生态文明建设。深入实施大气、水、土壤污染防治行动计划，加强生态保护和修复。今后五年，单位国内生产总值用水量、能耗、二氧化碳排放量分别下降23%、15%、18%，森林覆盖率达到23.04%，能源资源开发利用效率大幅提高，生态环境质量总体改善。特别是治理大气雾霾取得明显进展，地级及以上城市空气质量优良天数比率超过80%。我们要持之以恒，建设天蓝、地绿、水清的美丽中国。

　　——深化改革开放，构建发展新体制。发展根本上要靠改革开放。必须全面深化改革，坚持和完善基本经济制度，建立现代产权制度，基本建成法治政府，使市场在资源配置中起决定性作用和更好发挥政府作用，加快形成引领经济发展新常态的体制机制和发展方式。"一带一路"建设取得重大进展，国际产能合作实现新的突破。对外贸易向优进优出转变，服务贸易比重显著提升，从贸易大国迈向贸易强国。全面实行准入前国民待遇加负面清单管理制度，逐步构建高标准自由贸易区网络，基本形成开放型经济新体制新格局。

　　——持续增进民生福祉，使全体人民共享发展成果。坚持以人民为中心的发展思想，努力补齐基本民生保障的短板，朝着共同富裕方向稳步前进。坚决打赢脱贫攻坚战，我国现行标准下的农村贫困人口实现脱贫，贫困县全部摘帽，解决区域性整体贫困。建立国家基本公共服务项目清单。建立健全更加公平更可持续的社会保障制度。实施义务教育学校标准化、普及高中阶段教育、建设世界一流大学和一流学科等工程，劳动年龄人口平均受教育年限从10.23年提高到10.8年。实现城镇新增就业5000万人以上。完善收入分配制度，缩小收入差距，提高中等收入人口比重。完善住房保障体系，城镇棚户区住房改造2000万套。推进健康中国建设，人均预期寿命提高1岁。构建现代公共文化服务体系，实施公民道德建设、中华文化传承等工程。我们既要让人民的物质生活更殷实，又要让人民的精神生活更丰富。

　　做好"十三五"时期经济社会发展工作，实现全面建成小康社会目标，必须着力把握好三点。一是牢牢抓住发展第一要务不放松。发展是硬道理，是解决我国所有问题的关键。今后五年是跨越"中等收入陷阱"的重要阶段，各种矛盾和风险明显增多。发展如逆水行舟，不进则退。必须毫不动摇坚持以经济建设为中心，推动科学发展，妥善应对挑战，使中国经济这艘巨轮破浪远航。二是大力推进结构性改革。当前发展中总量问题与结构性问题并存，结构性问题更加突出，要用改革的办法推进结构调整。在适度扩大总需求的同时，突出抓好供给侧结构性改革，既做减法，又做加法，减少无效和低端供给，扩大有效和中高端供给，增加公共产品和公共服务供给，使供给和需求协同促进经济发展，提高全要素生产率，不断解放和发展社会生产力。三是加快新旧发展动能接续转换。经济发展必然会有新旧动能迭代更替的过程，当传统动能由强变弱时，需要新动能异军突起和传统动能转型，形成新的"双引擎"，才能推动经济持续增长、跃上新台阶。当前我国发展正处于这样一个关键时期，必须培育壮大新动能，加快发展新经济。要推动新技术、新产业、新业态加快成长，以体制机制创新促进分享经济发展，建设共享平台，做大高技术产业、现代服务业等新兴产业集群，打造动力强劲的新引擎。运用信息网络等现代技术，推动生产、管理和营销模式变革，重塑产业链、供应链、价值链，改造提升传统动能，使之焕发新的生机与活力。

　　从根本上说，发展的不竭力量蕴藏在人民群众之中。9亿多劳动力、1亿多受过高等教育和有专业技能的人才，是我们最大的资源和优势。实现新旧动能转换，推动发展转向更多依靠人力人才资源和科技创新，既是一个伴随阵痛的调整过程，也是一个充满希望的升级过程。只要闯过这个关口，中国经济就一定能够浴火重生、再创辉煌。

　　展望今后五年，我们充满必胜信心。如期实现全面建成小康社会目标，人民生活将会更加美好，中国特色社会主义事业前景一定会更加光明！

　　三、2016年重点工作

　　今年是全面建成小康社会决胜阶段的开局之年，也是推进结构性改革的攻坚之年。做好政府工作，必须高举中国特色社会主义伟大旗帜，全面贯彻党的十八大和十八届三中、四中、五中全会精神，以邓小平理论、"三个代表"重要思想、科学发展观为指导，深入贯彻习近平总书记系列重要讲话精神，按照"五位一体"总体布局和"四个全面"战略布局，坚持改革开放，坚持以新发展理念引领发展，坚持稳中求进工作总基调，适应经济发展新常态，实行宏观政策要稳、产业政策要准、微观政策要活、改革政策要实、社会政策要托底的总体思路，把握好稳增长与调结构的平衡，保持经济运行在合理区间，着力加强供给侧结构性改革，加快培育新的发展动能，改造提升传统比较优势，抓好去产能、去库存、去杠杆、降成本、补短板，加强民生保障，切实防控风险，努力实现"十三五"时期经济社会发展良好开局。

　　今年发展的主要预期目标是：国内生产总值增长6.5%-7%，居民消费价格涨幅3%左右，城镇新增就业1000万人以上，城镇登记失业率4.5%以内，进出口回稳向好，国际收支基本平衡，居民收入增长和经济增长基本同步。单位国内生产总值能耗下降3.4%以上，主要污染物排放继续减少。

　　经济增长预期目标6.5%-7%，考虑了与全面建成小康社会目标相衔接，考虑了推进结构性改革的需要，也有利于稳定和引导市场预期。稳增长主要是为了保就业、惠民生，有6.5%-7%的增速就能够实现比较充分的就业。

　　综合分析各方面情况，今年我国发展面临的困难更多更大、挑战更为严峻，我们要做打硬仗的充分准备。从国际看，世界经济深度调整、复苏乏力，国际贸易增长低迷，金融和大宗商品市场波动不定，地缘政治风险上升，外部环境的不稳定不确定因素增加，对我国发展的影响不可低估。从国内看，长期积累的矛盾和风险进一步显现，经济增速换挡、结构调整阵痛、新旧动能转换相互交织，经济下行压力加大。但困难和挑战并不可怕。中国的发展从来都是在应对挑战中前进的，没有过不去的坎。经过多年的快速发展，我国物质基础雄厚，经济韧性强、潜力足、回旋余地大，改革开放不断注入新动力，创新宏观调控积累了丰富经验。特别是我们有中国共产党的坚强领导和中国特色社会主义制度，中国人民勤劳智慧。只要我们万众一心，共克时艰，就一定能够实现全年经济社会发展目标。

　　今年要重点做好八个方面工作。

　　(一)稳定和完善宏观经济政策，保持经济运行在合理区间。我们宏观调控还有创新手段和政策储备，既要立足当前、有针对性地出招，顶住经济下行压力，又要着眼长远、留有后手、谋势蓄势。继续实施积极的财政政策和稳健的货币政策，创新宏观调控方式，加强区间调控、定向调控、相机调控，统筹运用财政、货币政策和产业、投资、价格等政策工具，采取结构性改革尤其是供给侧结构性改革举措，为经济发展营造良好环境。

　　积极的财政政策要加大力度。今年拟安排财政赤字2.18万亿元，比去年增加5600亿元，赤字率提高到3%。其中，中央财政赤字1.4万亿元，地方财政赤字7800亿元。安排地方专项债券4000亿元，继续发行地方政府置换债券。我国财政赤字率和政府负债率在世界主要经济体中相对较低，这样的安排是必要的、可行的，也是安全的。

　　适度扩大财政赤字，主要用于减税降费，进一步减轻企业负担。今年将采取三项举措。一是全面实施营改增，从5月1日起，将试点范围扩大到建筑业、房地产业、金融业、生活服务业，并将所有企业新增不动产所含增值税纳入抵扣范围，确保所有行业税负只减不增。二是取消违规设立的政府性基金，停征和归并一批政府性基金，扩大水利建设基金等免征范围。三是将18项行政事业性收费的免征范围，从小微企业扩大到所有企业和个人。实施上述政策，今年将比改革前减轻企业和个人负担5000多亿元。同时，适当增加必要的财政支出和政府投资，加大对民生等薄弱环节的支持。创新财政支出方式，优化财政支出结构，该保的一定要保住，该减的一定要减下来。

　　加快财税体制改革。合理确定增值税中央和地方分享比例。把适合作为地方收入的税种下划给地方，在税政管理权限方面给地方适当放权。进一步压缩中央专项转移支付规模，今年一般性转移支付规模增长12.2%。全面推开资源税从价计征改革。依法实施税收征管。建立规范的地方政府举债融资机制，对财政实力强、债务风险较低的，按法定程序适当增加债务限额。各级政府要坚持过紧日子，把每一笔钱都花在明处、用在实处。

　　稳健的货币政策要灵活适度。今年广义货币M2预期增长13%左右，社会融资规模余额增长13%左右。要统筹运用公开市场操作、利率、准备金率、再贷款等各类货币政策工具，保持流动性合理充裕，疏通传导机制，降低融资成本，加强对实体经济特别是小微企业、"三农"等支持。

　　深化金融体制改革。加快改革完善现代金融监管体制，提高金融服务实体经济效率，实现金融风险监管全覆盖。深化利率市场化改革。继续完善人民币汇率市场化形成机制，保持人民币汇率在合理均衡水平上基本稳定。深化国有商业银行和开发性、政策性金融机构改革，发展民营银行，启动投贷联动试点。推进股票、债券市场改革和法治化建设，促进多层次资本市场健康发展，提高直接融资比重。适时启动"深港通"。建立巨灾保险制度。规范发展互联网金融。大力发展普惠金融和绿色金融。加强全口径外债宏观审慎管理。扎紧制度笼子，整顿规范金融秩序，严厉打击金融诈骗、非法集资和证券期货领域的违法犯罪活动，坚决守住不发生系统性区域性风险的底线。

　　(二)加强供给侧结构性改革，增强持续增长动力。围绕解决重点领域的突出矛盾和问题，加快破除体制机制障碍，以供给侧结构性改革提高供给体系的质量和效率，进一步激发市场活力和社会创造力。

　　推动简政放权、放管结合、优化服务改革向纵深发展。以敬民之心，行简政之道，切实转变政府职能、提高效能。继续大力削减行政审批事项，注重解决放权不同步、不协调、不到位问题，对下放的审批事项，要让地方能接得住、管得好。深化商事制度改革，开展证照分离试点。全面公布地方政府权力和责任清单，在部分地区试行市场准入负面清单制度。对行政事业性收费、政府定价或指导价经营服务性收费、政府性基金、国家职业资格，实行目录清单管理。深化价格改革，加强价格监管。修改和废止有碍发展的行政法规和规范性文件。创新事中事后监管方式，全面推行"双随机、一公开"监管，随机抽取检查对象，随机选派执法检查人员，及时公布查处结果。推进综合行政执法改革，实施企业信用信息统一归集、依法公示、联合惩戒、社会监督。大力推行"互联网+政务服务"，实现部门间数据共享，让居民和企业少跑腿、好办事、不添堵。简除烦苛，禁察非法，使人民群众有更平等的机会和更大的创造空间。

　　充分释放全社会创业创新潜能。着力实施创新驱动发展战略，促进科技与经济深度融合，提高实体经济的整体素质和竞争力。一是强化企业创新主体地位。落实企业研发费用加计扣除，完善高新技术企业、科技企业孵化器等税收优惠政策。支持行业领军企业建设高水平研发机构。加快将国家自主创新示范区试点政策推广到全国，再建设一批国家自主创新示范区、高新区，建设全面创新改革试验区。二是发挥大众创业、万众创新和"互联网+"集众智汇众力的乘数效应。打造众创、众包、众扶、众筹平台，构建大中小企业、高校、科研机构、创客多方协同的新型创业创新机制。建设一批"双创"示范基地，培育创业服务业，发展天使、创业、产业等投资。支持分享经济发展，提高资源利用效率，让更多人参与进来、富裕起来。实施更积极、更开放、更有效的人才引进政策。加强知识产权保护和运用，依法严厉打击侵犯知识产权和制假售假行为。三是深化科技管理体制改革。扩大高校和科研院所自主权，砍掉科研管理中的繁文缛节。实施支持科技成果转移转化的政策措施，完善股权期权税收优惠政策和分红奖励办法，鼓励科研人员创业创新。大力弘扬创新文化，厚植创新沃土，营造敢为人先、宽容失败的良好氛围，充分激发企业家精神，调动全社会创业创新积极性，汇聚成推动发展的磅礴力量。

　　着力化解过剩产能和降本增效。重点抓好钢铁、煤炭等困难行业去产能，坚持市场倒逼、企业主体、地方组织、中央支持，运用经济、法律、技术、环保、质量、安全等手段，严格控制新增产能，坚决淘汰落后产能，有序退出过剩产能。采取兼并重组、债务重组或破产清算等措施，积极稳妥处置"僵尸企业"。完善财政、金融等支持政策，中央财政安排1000亿元专项奖补资金，重点用于职工分流安置。采取综合措施，降低企业交易、物流、财务、用能等成本，坚决遏制涉企乱收费行为。

　　努力改善产品和服务供给。突出抓好三个方面。一是提升消费品品质。加快质量安全标准与国际标准接轨，建立商品质量惩罚性赔偿制度。鼓励企业开展个性化定制、柔性化生产，培育精益求精的工匠精神，增品种、提品质、创品牌。二是促进制造业升级。深入推进"中国制造+互联网"，建设若干国家级制造业创新平台，实施一批智能制造示范项目，启动工业强基、绿色制造、高端装备等重大工程。落实加速折旧政策，组织实施重大技术改造升级工程。三是加快现代服务业发展。启动新一轮国家服务业综合改革试点，实施高技术服务业创新工程，大力发展数字创意产业。放宽市场准入，提高生产性服务业专业化、生活性服务业精细化水平。建设一批光网城市，推进5万个行政村通光纤，让更多城乡居民享受数字化生活。

　　大力推进国有企业改革。今明两年，要以改革促发展，坚决打好国有企业提质增效攻坚战。推动国有企业特别是中央企业结构调整，创新发展一批，重组整合一批，清理退出一批。推进股权多元化改革，开展落实企业董事会职权、市场化选聘经营者、职业经理人制度、混合所有制、员工持股等试点。深化企业用人制度改革，探索建立与市场化选任方式相适应的高层次人才和企业经营管理者薪酬制度。加快改组组建国有资本投资、运营公司。以管资本为主推进国有资产监管机构职能转变，防止国有资产流失，实现国有资产保值增值。赋予地方更多国有企业改革自主权。加快剥离国有企业办社会职能，解决历史遗留问题，让国有企业瘦身健体，增强核心竞争力。

　　更好激发非公有制经济活力。大幅放宽电力、电信、交通、石油、天然气、市政公用等领域市场准入，消除各种隐性壁垒，鼓励民营企业扩大投资、参与国有企业改革。在项目核准、融资服务、财税政策、土地使用等方面一视同仁。依法平等保护各种所有制经济产权，严肃查处侵犯非公有制企业及非公有制经济人士合法权益的行为，营造公平、公正、透明、稳定的法治环境，促进各类企业各展其长、共同发展。

　　(三)深挖国内需求潜力，开拓发展更大空间。适度扩大需求总量，积极调整改革需求结构，促进供给需求有效对接、投资消费有机结合、城乡区域协调发展，形成对经济发展稳定而持久的内需支撑。

　　增强消费拉动经济增长的基础作用。适应消费升级趋势，破除政策障碍，优化消费环境，维护消费者权益。支持发展养老、健康、家政、教育培训、文化体育等服务消费。壮大网络信息、智能家居、个性时尚等新兴消费。鼓励线上线下互动，推动实体商业创新转型。完善物流配送网络，促进快递业健康发展。活跃二手车市场，加快建设城市停车场和新能源汽车充电设施。在全国开展消费金融公司试点，鼓励金融机构创新消费信贷产品。降低部分消费品进口关税，增设免税店。落实带薪休假制度，加强旅游交通、景区景点、自驾车营地等设施建设，规范旅游市场秩序，迎接正在兴起的大众旅游时代。

　　发挥有效投资对稳增长调结构的关键作用。我国基础设施和民生领域有许多短板，产业亟需改造升级，有效投资仍有很大空间。今年要启动一批"十三五"规划重大项目。完成铁路投资8000亿元以上、公路投资1.65万亿元，再开工20项重大水利工程，建设水电核电、特高压输电、智能电网、油气管网、城市轨道交通等重大项目。中央预算内投资增加到5000亿元。深化投融资体制改革，继续以市场化方式筹集专项建设基金，推动地方融资平台转型改制进行市场化融资，探索基础设施等资产证券化，扩大债券融资规模。完善政府和社会资本合作模式，用好1800亿元引导基金，依法严格履行合同，充分激发社会资本参与热情。

　　深入推进新型城镇化。城镇化是现代化的必由之路，是我国最大的内需潜力和发展动能所在。今年重点抓好三项工作。一是加快农业转移人口市民化。深化户籍制度改革，放宽城镇落户条件，建立健全"人地钱"挂钩政策。扩大新型城镇化综合试点范围。居住证具有很高的含金量，要加快覆盖未落户的城镇常住人口，使他们依法享有居住地义务教育、就业、医疗等基本公共服务。发展中西部地区中小城市和小城镇，容纳更多的农民工就近就业创业，让他们挣钱顾家两不误。二是推进城镇保障性安居工程建设和房地产市场平稳健康发展。今年棚户区住房改造600万套，提高棚改货币化安置比例。完善支持居民住房合理消费的税收、信贷政策，适应住房刚性需求和改善性需求，因城施策化解房地产库存。建立租购并举的住房制度，把符合条件的外来人口逐步纳入公租房供应范围。三是加强城市规划建设管理。增强城市规划的科学性、权威性、公开性，促进"多规合一"。开工建设城市地下综合管廊2000公里以上。积极推广绿色建筑和建材，大力发展钢结构和装配式建筑，提高建筑工程标准和质量。打造智慧城市，改善人居环境，使人民群众生活得更安心、更省心、更舒心。

　　优化区域发展格局。深入推进"一带一路"建设，落实京津冀协同发展规划纲要，加快长江经济带发展。制定实施西部大开发"十三五"规划，实施新一轮东北地区等老工业基地振兴战略，出台促进中部地区崛起新十年规划，支持东部地区在体制创新、陆海统筹等方面率先突破。促进资源型地区经济转型升级。支持革命老区、民族地区、边疆地区、贫困地区发展。制定国家海洋战略，保护海洋生态环境，拓展蓝色经济空间，建设海洋强国。

　　(四)加快发展现代农业，促进农民持续增收。继续毫不放松抓好"三农"工作，完善强农惠农富农政策，深化农村改革，拓展农民就业增收渠道，着力提高农业质量、效益和竞争力。

　　加快农业结构调整。粮食连续增产，为稳定物价、改善民生提供了有力保障，但也面临库存大幅增加、市场价格下跌等问题。要引导农民适应市场需求调整种养结构，适当调减玉米种植面积。按照"市场定价、价补分离"原则，积极稳妥推进玉米收储制度改革，保障农民合理收益。要多措并举消化粮食库存，大力支持农产品精深加工，延伸农业产业链条；制定新一轮退耕还林还草方案，今年退耕还林还草1500万亩，这件事一举多得，务必抓好。积极发展多种形式农业适度规模经营，完善对家庭农场、专业大户、农民合作社等新型经营主体的扶持政策，鼓励农户依法自愿有偿流转承包地，开展土地股份合作、联合或土地托管。深化农村集体产权、农垦、集体林权、国有林场、农田水利、供销社等改革。

　　强化农业基础支撑。全面完成永久基本农田划定并实行特殊保护，加强高标准农田建设，增加深松土地1.5亿亩，新增高效节水灌溉面积2000万亩。探索耕地轮作休耕制度试点。加强农业科技创新与推广，深入开展粮食绿色高产高效创建，实施化肥农药零增长行动。保障财政对农业投入，建立全国农业信贷担保体系，完善农业保险制度，引导带动更多资金投向现代农业建设。

　　改善农村公共服务。加大农村基础设施建设力度，新建改建农村公路20万公里，具备条件的乡镇和建制村要加快通硬化路、通客车。抓紧新一轮农村电网改造升级，两年内实现农村稳定可靠供电服务和平原地区机井通电全覆盖。实施饮水安全巩固提升工程。推动电子商务进农村。建设美丽宜居乡村。

　　实施脱贫攻坚工程。今年要完成1000万以上农村贫困人口脱贫任务，其中易地搬迁脱贫200万人以上，继续推进贫困农户危房改造。中央财政扶贫资金增长43.4%。在贫困县推进涉农资金整合。坚持精准扶贫脱贫，因人因地施策。大力培育特色产业，支持就业创业。解决好通路、通水、通电、通网络等问题，增强集中连片特困地区发展能力。国家各项惠民政策和民生项目，要向贫困地区倾斜。深入开展定点扶贫、东西协作扶贫，支持社会力量参与脱贫攻坚。扶贫脱贫是硬任务，各级政府已经立下军令状，必须按时保质保量完成。

　　(五)推进新一轮高水平对外开放，着力实现合作共赢。面对国际经济合作和竞争格局的深刻变化，顺应国内经济提质增效升级的迫切需要，要坚定不移扩大对外开放，在开放中增强发展新动能、增添改革新动力、增创竞争新优势。

　　扎实推进"一带一路"建设。统筹国内区域开发开放与国际经济合作，共同打造陆上经济走廊和海上合作支点，推动互联互通、经贸合作、人文交流。构建沿线大通关合作机制，建设国际物流大通道。推进边境经济合作区、跨境经济合作区、境外经贸合作区建设。坚持共商共建共享，使"一带一路"成为和平友谊纽带、共同繁荣之路。

　　扩大国际产能合作。坚持企业为主、政府推动、市场化运作，实施一批重大示范项目。落实和完善财税金融支持政策，设立人民币海外合作基金，用好双边产能合作基金。推动装备、技术、标准、服务走出去，打造中国制造金字品牌。

　　促进外贸创新发展。面对外需持续低迷的严峻形势，要多措并举，遏制进出口下滑势头。一要加快落实和完善政策。优化出口退税率结构，确保及时足额退税，严厉打击骗取退税。增加短期出口信用保险规模，实现成套设备出口融资保险应保尽保。二要鼓励商业模式创新。扩大跨境电子商务试点，支持企业建设一批出口产品"海外仓"，促进外贸综合服务企业发展。三要优化贸易结构。开展服务贸易创新发展试点，增加服务外包示范城市，加快发展文化对外贸易。进一步整合优化海关特殊监管区域，促进加工贸易向中西部地区转移、向产业链中高端延伸。四要推进贸易便利化。全面推广国际贸易"单一窗口"。降低出口商品查验率。五要实施更加积极的进口政策。扩大先进技术设备、关键零部件及紧缺能源原材料进口。

　　提高利用外资水平。继续放宽投资准入，扩大服务业和一般制造业开放，简化外商投资企业设立程序，加大招商引资力度。创新内陆和沿边开放模式，打造新的外向型产业集群，引导外资更多投向中西部地区。扩大自贸试验区试点。创新开发区体制机制。我们将营造更加公平、更为透明、更可预期的投资环境，中国要始终成为富有吸引力的外商投资热土。

　　加快实施自由贸易区战略。积极商签区域全面经济伙伴关系协定，加快中日韩自贸区等谈判，推进中美、中欧投资协定谈判，加强亚太自贸区联合战略研究。我们愿与各方一道，推进贸易投资自由化，共同构建均衡、共赢、包容的国际经贸体系。

　　(六)加大环境治理力度，推动绿色发展取得新突破。治理污染、保护环境，事关人民群众健康和可持续发展，必须强力推进，下决心走出一条经济发展与环境改善双赢之路。

　　重拳治理大气雾霾和水污染。今年化学需氧量、氨氮排放量要分别下降2%，二氧化硫、氮氧化物排放量分别下降3%，重点地区细颗粒物(PM2.5)浓度继续下降。着力抓好减少燃煤排放和机动车排放。加强煤炭清洁高效利用，减少散煤使用，推进以电代煤、以气代煤。全面实施燃煤电厂超低排放和节能改造。加快淘汰不符合强制性标准的燃煤锅炉。增加天然气供应，完善风能、太阳能、生物质能等发展扶持政策，提高清洁能源比重。鼓励秸秆资源化利用，减少直接焚烧。全面推广车用燃油国五标准，淘汰黄标车和老旧车380万辆。在重点区域实行大气污染联防联控。全面推进城镇污水处理设施建设与改造，加强农业面源污染和流域水环境综合治理。加大工业污染源治理力度，对排污企业全面实行在线监测。强化环境保护督察。新修订的环境保护法必须严格执行，对超排偷排者必须严厉打击，对姑息纵容者必须严肃追究。

　　大力发展节能环保产业。扩大绿色环保标准覆盖面。支持推广节能环保先进技术装备，广泛开展合同能源管理和环境污染第三方治理。加大建筑节能改造力度，加快传统制造业绿色改造。开展全民节能、节水行动，推进垃圾分类处理，健全再生资源回收利用网络，把节能环保产业培育成我国发展的一大支柱产业。

　　加强生态安全屏障建设。健全生态保护补偿机制。停止天然林商业性采伐，实行新一轮草原生态保护补助奖励政策。推进地下水超采区综合治理试点，实施湿地保护与恢复工程，继续治理荒漠化、石漠化和水土流失。保护环境，人人有责。每一个社会成员都要自觉行动起来，为建设美丽中国贡献力量。

　　(七)切实保障改善民生，加强社会建设。为政之道，民生为本。我们要念之再三、铭之肺腑，多谋民生之利，多解民生之忧。财政收入增长虽放缓，但该给群众办的实事一件也不能少。

　　着力扩大就业创业。实施更加积极的就业政策，鼓励以创业带动就业。今年高校毕业生将高达765万人，要落实好就业促进计划和创业引领计划，促进多渠道就业创业。用好失业保险基金结余，增加稳就业资金规模，做好企业下岗职工再就业工作，对城镇就业困难人员提供托底帮扶。完成2100万人次以上农民工职业技能提升培训任务。加强对灵活就业、新就业形态的扶持。切实做好退役军人安置和就业创业服务工作。

　　发展更高质量更加公平的教育。教育承载着国家的未来、人民的期盼。公共教育投入要加大向中西部和边远、贫困地区倾斜力度。统一城乡义务教育经费保障机制，改善薄弱学校和寄宿制学校办学条件。鼓励普惠性幼儿园发展。办好特殊教育。大力发展现代职业教育，分类推进中等职业教育免除学杂费。对贫困家庭学生率先免除普通高中学杂费。落实提高乡村教师待遇政策。加快推进远程教育，扩大优质教育资源覆盖面。提升高校教学水平和创新能力，推动具备条件的普通本科高校向应用型转变。继续扩大重点高校面向贫困地区农村招生规模，落实和完善农民工随迁子女在当地就学和升学考试政策。支持和规范民办教育发展。从家庭到学校、从政府到社会，都要为孩子们的安全健康、成长成才担起责任，共同托起明天的希望。

　　协调推进医疗、医保、医药联动改革。健康是幸福之基。今年要实现大病保险全覆盖，政府加大投入，让更多大病患者减轻负担。中央财政安排城乡医疗救助补助资金160亿元，增长9.6%。整合城乡居民基本医保制度，财政补助由每人每年380元提高到420元。改革医保支付方式，加快推进基本医保全国联网和异地就医结算。扩大公立医院综合改革试点城市范围，协同推进医疗服务价格、药品流通等改革。深化药品医疗器械审评审批制度改革。加快培养全科医生、儿科医生。在70%左右的地市开展分级诊疗试点。基本公共卫生服务经费财政补助从人均40元提高到45元，促进医疗资源向基层和农村流动。鼓励社会办医。发展中医药、民族医药事业。建立健全符合医疗行业特点的人事薪酬制度，保护和调动医务人员积极性。完善一对夫妇可生育两个孩子的配套政策。为了人民健康，要加快健全统一权威的食品药品安全监管体制，严守从农田到餐桌、从实验室到医院的每一道防线，让人民群众吃得安全、吃得放心。

　　织密织牢社会保障安全网。继续提高退休人员基本养老金标准。各地要切实负起责任，确保养老金按时足额发放。制定划转部分国有资本充实社保基金办法。开展养老服务业综合改革试点，推进多种形式的医养结合。落实临时救助、特困人员救助供养等制度。城乡低保人均补助标准分别提高5%和8%。加快健全城乡社会救助体系，使困难群众遇急有助、遇困有帮，让社会充满关爱和温暖。

　　推进文化改革发展。用中国梦和中国特色社会主义凝聚共识、汇聚力量，培育和践行社会主义核心价值观，加强爱国主义教育。实施哲学社会科学创新工程，发展文学艺术、新闻出版、广播影视、档案等事业。建设中国特色新型智库。加强文化遗产保护利用。深化群众性精神文明创建活动，倡导全民阅读，普及科学知识，提高国民素质和社会文明程度。促进传统媒体与新兴媒体融合发展。培育健康网络文化。深化中外人文交流，加强国际传播能力建设。引导公共文化资源向城乡基层倾斜，推动文化产业创新发展。推进数字广播电视户户通。做好北京冬奥会和冬残奥会筹办工作，形成全民健身新时尚。

　　加强和创新社会治理。推进城乡社区建设，促进基层民主协商。支持工会、共青团、妇联等群团组织参与社会治理。加快行业协会商会与行政机关脱钩改革，依法规范发展社会组织，支持专业社会工作、志愿服务和慈善事业发展。完善社会信用体系。切实保障妇女、儿童、残疾人权益，加强对农村留守儿童和妇女、老人的关爱服务。开展法治宣传教育，做好法律援助和社区矫正工作。完善国家网络安全保障体系。创新社会治安综合治理机制，以信息化为支撑推进社会治安防控体系建设，依法惩治违法犯罪行为，严厉打击暴力恐怖活动，增强人民群众的安全感。改进信访、人民调解工作，有效化解矛盾纠纷，促进社会平安祥和。

　　生命高于一切，安全重于泰山。必须坚持不懈抓好安全生产和公共安全，加强安全基础设施和防灾减灾能力建设，健全监测预警应急机制，提高气象服务水平，做好地震、测绘、地质等工作。完善和落实安全生产责任、管理制度和考核机制，实行党政同责、一岗双责、失职追责，严格监管执法，坚决遏制重特大安全事故发生，切实保障人民生命财产安全。

　　(八)加强政府自身建设，提高施政能力和服务水平。重任千钧惟担当。面对异常艰巨复杂的改革发展任务，各级政府要深入贯彻落实新发展理念，把全面建成小康社会使命扛在肩上，把万家忧乐放在心头，建设人民满意的法治政府、创新政府、廉洁政府和服务型政府。

　　坚持依法履职，把政府活动全面纳入法治轨道。各级政府及其工作人员要严格遵守宪法和法律，自觉运用法治思维和法治方式推动工作，法定职责必须为，法无授权不可为。积极推行政府法律顾问制度。深入推进政务公开，充分发挥传统媒体、新兴媒体作用，利用好网络平台，及时回应社会关切，使群众了解政府做什么、怎么做。各级政府要依法接受同级人大及其常委会的监督，自觉接受人民政协的民主监督，接受社会和舆论监督，让权力在阳光下运行。

　　坚持廉洁履职，深入推进反腐倡廉。认真落实党风廉政建设主体责任，严厉整治各种顶风违纪行为。加强行政监察，推进审计全覆盖。以减权限权、创新监管等举措减少寻租空间，铲除滋生腐败土壤。推动党风廉政建设向基层延伸，坚决纠正侵害群众利益的不正之风，坚定不移惩治腐败。

　　坚持勤勉履职，提高执行力和公信力。政府工作人员要恪尽职守、夙夜在公，主动作为、善谋勇为。深入践行"三严三实"，增强政治意识、大局意识、核心意识、看齐意识，加强作风和能力建设，打造高素质专业化的公务员队伍。健全并严格执行工作责任制，确保各项政策和任务不折不扣落到实处。健全督查问责机制，坚决整肃庸政懒政怠政行为，决不允许占着位子不干事。健全激励机制和容错纠错机制，给改革创新者撑腰鼓劲，让广大干部愿干事、敢干事、能干成事。中国改革开放30多年的辉煌成就，就是广大干部群众干出来的。

　　上下同欲者胜。我们要充分发挥中央和地方两个积极性。对真抓实干成效明显的地方，在建设资金安排、新增建设用地、财政沉淀资金统筹使用等方面，加大奖励支持力度。鼓励各地从实际出发干事创业，形成竞相发展的生动局面。

　　各位代表！

　　中华民族是一个大家庭，促进各民族和睦相处、和衷共济、和谐发展，是各族人民的根本利益和共同责任。要坚持中国特色解决民族问题的正确道路，坚持和完善民族区域自治制度，严格执行党的民族政策，深入开展民族团结进步创建活动，推动建立各民族相互嵌入式的社会结构和社区环境，促进各民族交往交流交融。落实促进民族地区发展的差别化支持政策，保护和发展少数民族优秀传统文化及特色村镇，加大扶持人口较少民族发展力度，大力实施兴边富民行动，让全国各族人民共同迈向全面小康社会。

　　我们要全面贯彻党的宗教工作基本方针，坚持依法管理宗教事务，促进宗教关系和谐，发挥宗教界人士和信教群众在促进经济社会发展中的积极作用。

　　我们要认真落实侨务政策，依法维护海外侨胞和归侨侨眷的合法权益，充分发挥他们的独特优势和重要作用，不断增强海内外中华儿女的向心力。

　　各位代表！

　　过去一年，国防和军队建设取得显著成效。新的一年，要紧紧围绕实现党在新形势下的强军目标，深入推进政治建军、改革强军、依法治军，全面加强军队革命化现代化正规化建设，坚决维护国家安全。坚持党对军队绝对领导的根本原则和制度，落实古田全军政治工作会议精神。统筹推进各方向各领域军事斗争准备，严密组织日常战备和边海空防管控。加强后勤保障和装备发展。稳步推进领导指挥体制改革，部署展开军队规模结构和政策制度等改革。提高军队建设法治化水平。建设现代化武装警察部队。推动重要领域军民融合深度发展。加强国防动员建设。发展国防科技工业。各级政府要大力支持国防和军队建设，走出一条新时期鱼水情深的军政军民团结之路。

　　各位代表！

　　我们将全面准确贯彻"一国两制"、"港人治港"、"澳人治澳"、高度自治的方针，严格依照宪法和基本法办事。全力支持香港、澳门特别行政区行政长官和政府依法施政。发挥港澳独特优势，提升港澳在国家经济发展和对外开放中的地位和功能。深化内地与港澳合作，促进港澳提升自身竞争力。我们相信，香港、澳门一定会保持长期繁荣稳定。

　　我们要继续坚持对台工作大政方针，坚持"九二共识"政治基础，坚决反对"台独"分裂活动，维护国家主权和领土完整，维护两岸关系和平发展和台海和平稳定。推进两岸经济融合发展。促进两岸文教、科技等领域交流，加强两岸基层和青年交流。我们将秉持"两岸一家亲"理念，同台湾同胞共担民族大义，共享发展机遇，携手构建两岸命运共同体。

　　各位代表！

　　我们将继续高举和平、发展、合作、共赢的旗帜，践行中国特色大国外交理念，维护国家主权、安全、发展利益。办好在我国举行的二十国集团领导人峰会，推动世界经济创新增长，完善全球经济金融治理。加强与各主要大国协调合作，建设良性互动、合作共赢的大国关系。秉持亲诚惠容的周边外交理念，与地区国家持久和平相处、联动融合发展。深化南南合作、促进共同发展，维护发展中国家正当合法权益。建设性参与解决全球性和热点问题。加快海外利益保护能力建设，切实保护我国公民和法人安全。中国愿与国际社会一道，为人类和平与发展事业不懈努力！

　　各位代表！

　　奋斗才能赢得未来。让我们更加紧密地团结在以习近平同志为总书记的党中央周围，凝心聚力，奋发进取，努力完成今年经济社会发展目标任务，确保全面建成小康社会决胜阶段良好开局，为建成富强民主文明和谐的社会主义现代化国家、实现中华民族伟大复兴的中国梦作出新的贡献！
